# The Bayesian Design of Adaptive Clinical Trials

**DOI:** 10.3390/ijerph18020530

**Published:** 2021-01-10

**Authors:** Alessandra Giovagnoli

**Affiliations:** Department of Statistical Sciences, University of Bologna, 40126 Bologna, Italy; alessandra.giovagnoli@unibo.it

**Keywords:** adaptive designs, adaptive randomization, Bayesian designs, clinical trials, predictive power, target allocation

## Abstract

This paper presents a brief overview of the recent literature on adaptive design of clinical trials from a Bayesian perspective for statistically not so sophisticated readers. Adaptive designs are attracting a keen interest in several disciplines, from a theoretical viewpoint and also—potentially—from a practical one, and Bayesian adaptive designs, in particular, have raised high expectations in clinical trials. The main conceptual tools are highlighted here, with a mention of several trial designs proposed in the literature that use these methods, including some of the registered Bayesian adaptive trials to this date. This review aims at complementing the existing ones on this topic, pointing at further interesting reading material.

## 1. Introduction

This paper is a bird’s eye view of the recent literature on adaptive designs of clinical trials from a Bayesian perspective. Statistics plays a prominent role in the design as well as the analysis of the results of a clinical study and Bayesian ideas are well received by clinicians. In their book, Spiegelhalter and his coauthors [1] make a strong case in favour of Bayesian methods in health care, and in the last two decades Bayesian statistics has had a large impact in the medical field (see the superb review by Ashby [2]), the more so as its implementation gets easier thanks to better computational facilities. “Bayesian clinical trials: no more excuses” is the title of an editorial in Vol 6(3) of *Clinical Trials* [3]. The Bayesian approach has a good reputation at producing scientific openness and honesty.

The Bayesian paradigm is especially appropriate at the planning stage of a clinical trial, when external information, such as historical data, findings from previous studies, and expert opinions, is often available and awaiting to be made the most of. As Donald Berry and his colleagues state in [4], we are all Bayesian at the design stage! Health authorities have issued important statements on the statistical, clinical and regulatory aspects of Bayesian clinical trials ([5,6]), recently allowing and even advocating the use of innovative methods, in particular adaptive design; as the editors of this Special Issue point out, most statistical and biomedical journals have recently hosted proposals of trial designs with a Bayesian slant, in some cases virtual re-executions of published trials. A search carried out in PubMed in August 2020 has returned nearly 300 publications (half of them published in the last decade) which either propose or use Bayesian adaptive methods in the design of a clinical trial. This may be also thanks to the popularization by Donald Berry [7,8,9,10,11] and the efforts made by statisticians working in the pharmaceutical industry, one of the main players in the design of clinical trials, to incorporate Bayesian methods. This is shown in leading journals in clinical trial methodology, like *Pharmaceutical Statistics*, *The Journal of Biopharmaceutical Statistics* or *Biometrical Journal*.

Some confusion occasionally arises between the concepts of “Bayesian” and of “adaptive” design, because of similarities in the outlook: in the Bayesian paradigm, accrued data are used to update the prior distribution on the parameters, via Bayes’ Theorem, and in response-adaptive experiments the accrued data are used at each step, namely after each observation or predefined group of observations, to update the next planning decision. Either approach (Bayesian or adaptive) can stand on its own, and has developed independently of the other: we clarify this point later.

We are interested in trial designs that are both Bayesian and adaptive. The data are recursively evaluated during the experiment: the posterior parameter distribution is recursively updated and used to modify the execution of the trial according to *a previously established rule*. The textbook by Berry, Carlin, Lee, and Muller [12] successfully illustrates Bayesian adaptive methods in clinical research and deals with design issues too. It goes almost without saying that randomization is a must in a clinical trial (for Bayesians too), to counteract several types of bias, for instance selection bias.

The subject is advancing fast in many directions and it is impossible to keep up with all the different threads. Dealing with this topic properly would require several books. In this paper it has been chosen to focus on the methodology, followed by several examples of instances in which the methods are applied. The subject matter is organized as follows: Section 2 is a general discussion of Bayesian designs. In Section 3 we summarize the theory of response-adaptive designs from the *frequentist* viewpoint, with emphasis on their importance in clinical studies. Moving on to Bayesian adaptive trial designs, Section 4 deals with the different methodological approaches (utility-based, probability-only, predictive) and their use for randomization, sample size determination and early stopping. Section 5 reports examples from the literature. Section 6 lists some well-known real life trials performed according to a Bayesian adaptive design. In Section 7 we mention the on-going debate on the relevance of response-adaptive randomization designs in clinical trials, although at present the controversy is not directly addressed to Bayesian methods. Conclusions are drawn in Section 8.

## 2. Bayesian Designs

To start with, the very meaning of the term “Bayesian design” has to be contextualized. It is important to specify whether the Bayesian approach relates just to the pre-experimental plan, or to the analysis of the data as well. Because “Bayesian” is sometimes taken to mean “subjective”, for clinical trials an “objective” data analysis is often preferred. In clinical trials in particular the operating characteristics of the design often include the power of some statistical test, strictly speaking a non-Bayesian feature. So some studies are hybrid: the inference is frequentist, but a prior probability on the parameters is used at the design stage. Several authors, for example Ventz et al. [13] have presented ways of combining Bayesian models and frequentist analyses in clinical trials. At the start, design issues were neglected by Bayesian statisticians, but Bayesian design has latterly become a well-established subdiscipline, as shown by the outstanding number of quotations of Chaloner and Verdinelli’s [14] paper throughout the years. Unfortunately, that review has never been updated, and does not mention the topic of adaptive design, which at the time was only beginning to appear in the literature.

As is well known, in the Bayesian model-based paradigm, the parameters of the model are treated as random quantities and the a priori knowledge about unknown effects of the interventions is expressed by means of a prior probability distribution on the model parameters. In the non-adaptive case, the experiment is designed entirely before the new data are acquired, and its performance may depend on the data still to be observed The fully Bayesian way to designing the trial would be a utility-based one, in accordance with Lindley [15]. In the model-based approach, the utility U will be a function of the experimental plan, the data *y*, and the model parameter values θ. A design action (deterministic or randomized) is chosen so as to maximize the expectation of the utility function with respect to the joint distribution of the parameters *and* the future data.

In the eighties Smith and Verdinelli [16], Giovagnoli and Verdinelli [17] and Chaloner [18] proposed Bayesian designs that maximize the precision of the posterior distribution of the treatment effects under a linear statistical model. For non-linear models, Chaloner and Larntz [19] suggested choosing the design that optimizes the expectation, with respect to the parameters’ prior, of one of the well-known design criteria: the best known ones—the so-called D-optimality and trace-optimality for instance (“Optimal design” [20])—have also Bayesian interpretations. Since optimality criteria are functions of the Fisher information matrix, which is already an average over the possible responses, this approach too is not conditional on the data. This suggestion has become very popular and keeps being used in the literature, with many variants, not all of which are entirely convincing. 

It should be stressed that the choice of a suitable utility function is related to the objectives of the trial. The specification of the function U is certainly demanding, as various aspects of the decision context have to be formally quantified. The main purpose of any experiment is obviously inference. Even if the subjects are patients, the main goal of a trial is knowledge, not healthcare, so the expected gain in information is a relevant component of U. In the past, designs for Bayesian analysis of the data would mostly choose utility functions based on Shannon information or on a quadratic loss. Nowadays, it is not uncommon to find Bayesian designs motivated by the precision of the estimates and power of the tests, in view of the fact that the experimental data will be analysed by frequentist tools. On the other hand, in a clinical trial the utility function may also need to reflect care for the subjects involved in the experiment. An opposite, rather extreme, example in the binary response case is the 0–1 utility: the gain is 1 for success, 0 for failure, which takes no account of inference. Since a clinical trial is a multi-objective experiment, the utility will more likely be a trade-off between several purposes: ethical, inferential and possibly economic. Verdinelli and Kadane [21] suggested a utility function which is a linear combination of the total expected value of future observations and the Shannon’s information of the experiment. Clyde and Chaloner [22] too have proposed weighted combinations of utility functions, which are still mathematically valid utilities. In this way optimal designs for multiple objectives can be handled in the decision-theoretic framework, so long as the utilities are standardized to a comparable scale. For instance with binary outcomes one of the usual optimality criteria—determinant or trace, standardized—can be combined with the percentage of expected successes. Clyde and Chaloner [22] show that this approach is equivalent to finding the optimal experiment under one (primary) criterion subject to constraints on the minimal efficiency of the experiment under criteria for other objectives. The weights will be chosen by the experimenter to reflect the relative importance given to each utility component: it is advisable that the ethical and/or economic impact should not completely outweigh the inferential goal.

Bayesian non-adaptive design has had numerous applications in clinical trials up to the end of the nineties and beyond, as shown in the paper by Ashby [2].

## 3. Adaptive Designs: The Frequentist Approach

Response-adaptive—or simply adaptive—experiments are those in which the accrued data and the past allocations are used at each step to choose how to perform the next observations, according to pre-established rules. In statistics, “response-adaptive” (sometimes also “flexible”) is a technical term. It means a predefined step-wise sequential algorithm which prescribes the modalities of the next step as a (deterministic or probabilistic) function of the accrued data. The choice of the pre-established rule is an essential part of the design—as indicated by the FDA [6]— and must be taken into account to draw correct inferences. Response-adaptive design does not mean simply that at any stage you may want to change your plans. It should be stressed that the updating process of an experiment cannot take place in a haphazard manner, which could undermine the ensuing statistical analysis of the data. Typically it could lead to miscalculating the variance of the estimates or to drawing wrong conclusions. Burman and Sonesson [23] give a very clear-minded discussion. Thus the correct design of adaptive experiments poses challenging statistical problems: because of the dependence on the past data, the design itself will be a stochastic process, whose realization is the sequence of actual assignments of the experiment.

A good introduction to adaptive designs is Chapter 1 of the collective volume edited by Pong and Chow [24]. It is useful to distinguish among the different types of adaptation useable in clinical trials:

(1) Adaptive allocation rule—change in the randomization procedure to modify the allocation proportion.
(2) Adaptive sampling rule—change in the number of study subjects (sample size) or change in study population: entry criteria for the patients change.
(3) Adaptive stopping rule—during the course of the trial, a data-dependent rule dictates whether and when to stop for harm/futility/efficacy.
(4) Adaptive enrichment: during the trial, treatments are added or dropped.

A more detailed classification is the one by Huskins et al. [25]. All types of adaptation may also take into account covariates, i.e., the patient’s prognostic factors/biomarkers. Adaptation may aim to ensure approximate balance of categorical covariates between the treatment arms in randomization (minimization and stratified randomization methods) or to best suit the characteristics of the subjects in the trial, when the covariates are of the “predictive” type, namely they interact with the treatments.

Despite the intricacies of the theory, including whether inference should be conditional on the design or unconditional as Baldi-Antognini and Giovagnoli point out [26], adaptive methods are attractive to experimenters: they look and sometimes are more flexible, efficient, ethical and/or economical, and proceeding sequentially seems to reflect real life practice. Among the possible applications, at present the clinical ones are playing a prominent role, to the point that response-adaptive designs are almost identified with clinical trials, notwithstanding their relevance in other disciplines: experimental economics, computer science, aerodynamics, combustion engineering, psychology, social sciences, and many others, as a simple search in Google Scholar easily shows. Altogether, adaptive design seems to be a more congenial perspective for practicing clinicians. Adaptive procedures are often feasible in clinical trials, in which typically the recruitment of subjects takes place sequentially, especially when they are healthy volunteers. In particular, they are fairly frequent in early phase studies, because of the inherently adaptive nature of such trials. The “steps” may consist of just one observation at-a-time, or more than one, as explained earlier. They may also represent natural stages of the experiment. Even a two-stage trial may be regarded as adaptive, if it is ruled in advance what decisions will be made conditional on the first stage data. Clearly such step-by-step procedures must be described and justified in the study protocol. A review of the literature unfortunately shows that some trials described as adaptive report not-so-rigorous ad-hoc adjustments of the experimental design along the way.

A quick overview of non-Bayesian adaptive designs methods may be helpful, to present the main ideas. An authoritative starting point is Rosenberger’s 1996 review [27] but important methodological advances have been made since.

### 3.1. Dose Finding

Trials for new drug development involve dose-finding. In Phase I we are usually interested in finding which value of the dosage *x* will produce a prescribed mean response, typically a small tolerated amount of toxicity. The experimental problem is essentially adaptive by its very nature: after each observation a decision has to be taken as to whether to leave the dosage unaltered for the next cohort or change it. There are the so-called rule-based designs without assumptions on the dose-toxicity response curve, like the notorious 3 + 3 rule. I have chosen not to dwell on these non-Bayesian methods, since a description can be easily found in several books about statistical methods in clinical trials. Another non parametric method is the Up-and-Down design, in which the assignment rule of the next patient involves a random component: see for instance Baldi-Antognini and Giovagnoli [26] for the theoretical properties. Surprisingly, only few developments of the Up-and-Down incorporating Bayesian ideas have taken place.

In the parametric set-up, the mean response is modelled as a smooth increasing function Q(x, θ) of the dose, depending on an unknown vector parameter θ. Frequent choices for Q(x, θ) are the logistic quantile function and the probit function. The parameter θ can be recursively estimated through Maximum Likelihood. The design problem is how to modify the doses efficiently each time.

Adaptive ideas show their potential also in “seamless” designs for Phase I and Phase II simultaneously ([28]), or Phase II together with Phase III ([29]).

### 3.2. Response-Adaptive Randomization

When equal allocation to the treatment arms was regarded as optimum, the only accepted randomization rule with two treatments was “tossing a fair coin”. Since Efron ([30]), randomization schemes that move away from the traditional equal allocation have gained consent. Efron’s *Biased Coin* is a randomization rule skewed in favour of the under-represented treatment(s), regardless of the data; response-adaptive randomization, on the other hand, is a “biased coin” that skews the allocations using the accrued data, usually to favour the best performing treatment. An important introduction to the mathematics of response adaptive randomization in clinical trials is the book by Hu and Rosenberger [31].

For two treatments *A* and *B* with binary responses and p_A_ and p_B_ success probabilities, basic methods are the well-known Play-the-Winner and Randomized Play-the-Winner: see the book by Rosenberger and Lachin [32] for details and also for recent theoretical developments in adaptive randomization. The modern approach consist in first choosing an ideal allocation proportion of the treatments—a “target”—obtained by a trade-off between several purposes: ethical, inferential and possibly economic, as suggested by Baldi-Antognini and Giovagnoli [33]: see also [34]. Then an adaptive, possibly randomized, procedure is devised with the property of converging to this target allocation for all values of the unknown model parameters. Suggested adaptive rules that achieve this purpose are the Sequential Maximum Likelihood design, the Doubly-adaptive Biased Coin Design and the Efficient Randomized Adaptive Design (ERADE), which is a response-adaptive version of Efron’s Biased Coin rule. These are explained, for instance, in Chapter 1 of the collective book edited by Sverdlov [35] that contains a description of the state-of-the art in adaptive randomization in clinical trials. The other contributions in the same volume dwell on further developments.

### 3.3. Sequential Monitoring

Selecting the sample size is the number one design concern in almost all experiments. In frequentist statistics the sample size is usually calculated so as to achieve a prescribed power for the statistical test of interest, under reasonable assumptions for the true state of nature. An adaptive approach to this problem is typically applied in clinical trials, when the trial design is in two or more stages. At the end of each stage the appropriate sample size for the next stage gets re-estimated, making use of the accrued data.

The sample size issue is also related to early stopping, since as is well-known in clinical trials another approach is to fix a (maximum) number for the sample and include the possibility to stop earlier, if certain conditions are met. Interim analyses of the data at predetermined time periods are conducted before the data collection has been completed, in order to stop the trial early if some of the treatments tested show to be clearly harmful or clearly useless, or obviously superior. This is the oldest practice in adaptive design, usually referred to as *group-sequential*. Decision rules for stopping consist in setting boundaries for a predetermined test statistic so that some error probability requirements are satisfied. There is a rich literature on this topic [36,37,38,39]. Adaptive early termination has been applied, for instance, in the recent 2020 World Health Organization Solidarity Trial for COVID-19 [40]. 

In bio-pharmaceutical experiments the attention has focussed in particular on stochastic curtailment ([41]). The stochastic curtailment rule computes the conditional power, i.e., the conditional probability that the summary statistics at the end of the trial is in the rejection region, given the current data available, under the null hypothesis of no effect or the alternative hypothesis (a clinically significant difference). This approach is based on a “prediction”, thus it leans towards the Bayesian philosophy.

Group-sequential designs in general do not consider treatment allocations other than equal size randomization. For some models, however, Jennison and Turnbull [42] have shown that response-adaptive randomization can be incorporated into a general family of group sequential tests without affecting the error probabilities. Zhu and Hu [43] have studied both theoretical and finite sample properties of designs which combine sequential monitoring with response-adaptive randomization.

## 4. The Bayesian Viewpoint in Response-Adaptive Designs

Adaptive designs are (deterministic or randomized) rules that at each stage of the trial, conditionally on the accrued data, prescribe how to choose the treatment assignments and/or include new or drop old treatments and/or choose the sample size for the next experimental stage and/or choose the next patients, and/or decide whether to stop the trial. In the Bayesian set-up, in order to choose the next step’s or stage’s settings in an optimal way the design rule makes use of the posterior distribution of the parameters, updated each time. If the approach is decision-based, the design rule recursively optimizes the posterior expected utility. Posterior distributions may also be used in more direct ways in the design of the experiment, without the decision theoretic framework, as we shall see in Section 4.1 about adaptive randomization. In the binary case, posterior probabilities correspond to the choice of the simplistic utility, where the gain is 1 for success, 0 for failure. An essential Bayesian tool is also the predictive probability of yet unobserved events, conditional on past data. Predictive distributions are useful in many adaptive design contexts, like trial monitoring and also deciding whether to conduct a future trial. Their use is shown in many clinical contexts in the book by Berry et al. [12].

Recently, Bayesian methods are more and more to be found naturally embedded in most of the emergent adaptive design ideas: the Continuous Reassessment Method for dose-finding in Phase I (see Section 5) is a typical example. Important review articles are by Chevret [44], who searched adaptive Bayesian design in the medical literature up to 2010, and by Rosner [45], who deals with Bayesian adaptive design in drug development.

Bayesian adaptive designs are sometimes called BAD, not a very exciting acronym! When the randomization rule is adaptive, they are called Bayesian Adaptive Randomization (BAR), Bayesian RAR or Bayesian Response-Adaptive Randomization (BRAR); we prefer Bayesian Adaptively Randomized Designs (BARDs), a much more inspiring acronym. In the literature there is occasionally a temptation to describe as Bayesian some adaptive designs that make no use of priors or posteriors, like the Bayesian Biased Coin Design by Atkinson and Biswas [46].

### 4.1. Bayesian Adaptive Randomization

The need to randomize treatments to patients is mandatory in all clinical trials, and is used in each Phase whenever possible, although it tends to be studied with particular reference to Phase III: these are multicenter case-control studies on large patient groups (300–3000 or more), aimed at assessing how effective the proposed new intervention(s) is in comparison with the current “gold standard”.

In spite of the popularity of Bayesian adaptive methods, Bayesian adaptive randomization for clinical trials does not seem to have been investigated extensively, as pointed out in the book by Atkinson and Biswas [47]. There is no straightforward Bayesian equivalent of Play-the-Winner for the case of binary data and two treatments, which is not surprising since Play-the-Winner is a myopic strategy, based on the most recent result, whereas the Bayesian paradigm is characterized by its use of all the past data.

The very early paper by Thompson [48] is worth a special mention, because its Bayesian way to randomization in the binary model—reminiscent of the Randomized Play-the-Winner, and called “probability only”—is still very popular to this day.

*“If P is the probability estimate (meaning the posterior probability) that one treatment is better than a second, as judged by data at present available, then we might take some monotone increasing function of P, say f(P), to fix the fraction of such individuals to be treated in the first manner until more evidence may be utilised, where 0 ≤ f(P) ≤ 1; the remaining fraction of such individuals (1 − f(P)) to be treated in the second manner; or we may establish a probability of treatment by the two methods of f(P) and 1 − f(P), respectively”*.

(Thompson, [48])

When *f*(P) = P this method is referred to as *randomized probability matching:* it is ethical from the patients’ viewpoint but pays no attention to inference. However the function *f* may be chosen so as to act as a stabilizing transformation, to avoid excess variability which has a negative effect on the inferential efficiency. It has become customary to take *f*(P) and 1 − *f*(P) proportional to [Pr(*p*_A_ > *p*_B_ | *y*)]^υ^ and [Pr(*p*_B_ > *p*_A_ | *y*)]^υ^, respectively, where υ is a positive quantity that modulates the tradeoff between the exploration (gaining information) and the exploitation (benefit for the patients) aims of the experiment. The value of υ recommended by Thall and Wathen [49], based on empirical experience, is υ = ½, or υ = n/2N, where n is the present sample size and N the total proposed one. Extensions of the theory allow the progressive reassessment of υ based on interim analysis data.

In principle, the idea of adaptive randomized designs converging to a prescribed target can be used in a Bayesian context as well. A utility function is chosen and at each step, a “temporary” target is found by optimizing the posterior expected utility. This pseudo-target—which is conditional on the data and gets updated each time—will be used, possibly after a suitable transformation, as the allocation probability of the randomization scheme. The intuitive meaning is to try to allocate the treatments—at each step—in the way that is optimal according to the present information. It may be looked at as a Bayesian equivalent of the frequentist Sequential Maximum Likelihood design mentioned in Section 3.2.

### 4.2. Sample Size Determination and Early Stopping

Although for a Bayesian analysis of the data in principle there is no need for preplanned sample sizes, nevertheless Bayesian design does consider sample size selection, both for practical reasons and for a potential classical inferential analysis. The prior distribution of the unknown quantities may be incorporated into finding the appropriate sample size in more ways than one (utility-based, pre-posterior, ecc.); see for instance [50]. Instead of the conditional power, the predictive power is often used, namely the predictive probability of rejecting the null hypothesis of no effect, or of no difference among effects: this approach indicates how the sample size of a clinical trial is to be adjusted so as to claim a success at the conclusion of the trial with an expected probability. The same ideas can be used when the trial is planned to be performed adaptively; at the end of each step the sample size of the next step will be selected.

As to sequential monitoring, Donald Berry [51] and Spiegelhalter and Freedman [52] were perhaps the very first to suggest the application of Bayesian tools for the decision to stop the trial before the planned sample size is reached. Monitoring can be based on the posterior probabilities of the hypotheses of interest, like the posterior probability that the treatment benefit lies above or below some boundary, or based on the predictive probabilities of the consequences of continuing the study; the predictive power, namely the expectation of the power function with respect to the distribution of the true underlying effect size, is often relevant when deciding on whether to stop a clinical trial for futility. Useful references are [53,54]. Alternatively, the decision of whether to interrupt the trial may derive from a proper utility function that quantifies the gain associated with the consequences of stopping or continuing. A good discussion of the appropriateness of the different Bayesian decision rules is found in the books by Spiegelhalter et al. [1] and Berry et al. [12], and in [55].

## 5. Suggestions of Bayesian Adaptive Design from the Literature

The best known Bayesian adaptive rule is the Continual Reassessment Method (CRM) by O’Quigley et al. [56,57] for Phase I: Cheung [58] devotes an entire book to it. It is aimed at finding a given quantile of the dose-response function Q(x, θ), with θ the unknown vector parameter. Often the response is toxicity and we are looking for the dose x_p_* corresponding to a given maximum probability p* of toxicity, called Maximum Tolerated Dose (MTD). Given a prior on θ, the expected value of x_p_* is calculated and the next set of observations is taken at the dose level nearest to it. The process is then iterated recusively and it can be proved to converge to the MTD. The main advantage of this design is that the majority of observations are centered around the dosage of interest. 

Many variants of the CRM have appeared over the years to handle different clinical scenarios, such as separate groups or late-onset toxicity. In particular:

- The TITE-CRM by Cheung and Chappell [59] incorporates the time-to-event of each patient allowing patients to be entered in a staggered fashion.
- Escalation with Overdose Control (EWOC) by Babb and Rogatko [60]: it is the same as CRM, except for the use of the αth-quantile of the MTD’s posterior, instead of its mean, when selecting the next dose. This allows rapid dose escalation while controlling the probability of exceeding MDT. The extension of EWOC to covariate utilization permits personalization of the dose level for each specific patient.
- The STARPAC design [61] uses a traditional rule-based design until the first patient has a dose limiting toxicity and then switches to a modified CRM.
- Yin and Yuan [62] use the rather controversial idea of averaging the statistical model with respect to the parameter prior in conjunction with the Continuous Reassessment Method.

Still about dose-finding trials of Phase I with a binary toxicity endpoint, examples of Bayes design rules obtained in a decision theoretic framework are:

- The modified Toxicity Probability Interval (mTPI) design [63]. The decision to escalate or de-escalate the dose is made by partitioning the probability interval into three subintervals. The posterior probability that p* is in each subinterval is calculated, divided by the width of the subinterval. The interval with the highest posterior probability mass dictates the dose decision for the next patient. The mTPI possesses desirable large- and small-sample properties. These designs are compared in a numerical study in [64].
- The Adaptive Bayesian Compound Design by McGree et al. [65]: the authors use a compound utility functions to account for the dual experimental goals of estimating the MTD and addressing the safety of subjects.

Bayesian optimal design theory is used adaptively in a two-stage Phase I design by Haines et al. [66].

There has been a widespread use of Bayesian response-adaptive randomization methods.

➢ Thompson’s idea for adaptive randomization, extended from the case of two treatment arms to several arms, has been applied by Thall, Inoue and Martin [67] to the design of a lymphocyte infusion trial.
➢ Under a beta-binomial model, Yuan, Huang and Liu [68] design a trial for leukemia. The randomization assigns an incoming patient to the treatment arm such that the imbalance of a prognostic score across the treatments is minimized. This score depends on an unknown parameter whose posterior mean is continuously updated during the ongoing trial.
➢ Still for the Beta Binomial model, in Giovagnoli [69] the trace criterion is used as the utility function and a recursive “biased coin” is found that maximizes the posterior utility. The sequential randomized treatment allocation is shown to converge to Neyman’s classical target, namely the optimal one according to the trace criterion.
➢ Under the same model, Xiao et al. [70] have defined a Bayesian Doubly-adaptive Biased Coin Design, using the posterior probabilities of p_A_ > p_B_ and of p_B_ > p_A_, for the target and an assignment rule similar to the ERADE mentioned in Section 3.2. They derive some asymptotic properties of their Bayesian design, namely convergence and asymptotic normality of the allocation proportion.
➢ Giovagnoli and Verdinelli [71] choose a recursive target that optimizes the posterior expectation of a compound utility function and the ERADE algorithm for convergence.

Turning to sample size determination and early stopping, sequential stopping is mainly associated with Phase II trials, but as early as 1994, Thall and Simon [72] developed a design with continuous monitoring until a high posterior probability is achieved that a drug is promising or that it is not promising, or until reaching the maximum sample size. This idea has been further refined and modified by a multitude of authors.

- Wang [73] predicts how the sample size of a clinical trial needs to be adjusted so as to claim a success at the conclusion of the trial with an expected probability.
- An interesting evaluation paper is by Uemura et al. [74].
- Continuous monitoring by means of predictive probabilities is given by Lee and Liu [75]: for the binary case, under a beta-binomial model, and a given maximum sample size, they recursively calculate the predictive probability of concluding the study rejecting the hypothesis of no efficacy of the new treatment. They search for the design parameters within the given constraints such that both the size and power of the test can be guaranteed.
- Yin, Chen and Lee [76] have coupled Thompson’s adaptive randomization design with predictive probability approaches for Phase II.
- Zhong et al. [77] introduce a two-stage design with sample size re-estimation at the interim stage which uses a fully Bayesian predictive approach to reduce an overly large initial sample size when necessary.

Decision-theoretic methods have been applied in this context too, for example by Cheng and Shen [78] for a comparative two-armed clinical trial. They specify a loss function, based on the cost for each patient and the costs of making incorrect decisions at the end of the trial. At each interim analysis, the decision to terminate or to continue the trial is based on the expected loss function while concurrently incorporating efficacy, futility and cost. The maximum number of interim analyses is determined adaptively by the observed data.

## 6. Bayesian Adaptive Designs in Registered Trials

Adaptive designs are mathematically sophisticated instruments. Their development is fairly recent, and the split that can be observed between theory and practice is not at all surprising. There are several obstacles—both technical and practical—to launching an adaptive trial, beyond the significant time and effort required by any clinical trial. Among other things, adaptive design requires updating information on accrued data, the speed of acquisition may be highly variable so there is the need to identify short-term endpoints that can be used to accurately predict treatment responses such as long-term mortality in terms of a gold-standard endpoint. The steps required to establish this type of design in a novel context are indeed fairly complex, as some case studies show (see for instance Mason et al. [79]. As to the Bayesian approach, this may include specialized software programs to run the study design, only made possible by recent advancements in computational algorithms and computer hardware ([80]).

Nevertheless, it is worth remarking that the philosophy of Bayesian adaptive designs has already made its way into the clinic. They are now fairly well established in cancer research ([10]), and to a lesser extent, in other clinical areas. As well as single study designs, Bayesian adaptive methods are being employed to build “platform” designs (Adaptive Platform Trials). These are trials for simultaneous testing of multiple treatment strategies in separate groups, with plans to discontinue any group that is definitively inferior at planned interim analyses. Trial patients are enrolled in a continuous manner via a common master protocol, with interventions entering and leaving the platform on the basis of a predefined decision algorithm. Several Adaptive Platform Trials are now funded in various disease areas (see Angus et al. [81], Brown et al. [82] and Talisa et al. [83] for a discussion).

The following is a non-exhaustive list of recent or still on-going clinical trials that incorporate Bayesian adaptive design features:

- The Randomized Embedded Multifactorial Adaptive Platform Trial in Community Acquired Pneumonia (REMAP-CAP): see [84]. It has set-up a sub-platform called “REMAP−COVID” on which the evaluation of specific treatments for COVID-19 is run.
- Anti-Thrombotic Therapy to Ameliorate Complications of COVID-19 (ATTACC) (see [85]), similar in purpose to RECAP-COVID.
- GBM AGILE, an adaptive clinical trial to deliver improved treatments for glioblastoma, now open and enrolling patients ([86]).
- STURDY, a randomized clinical trial of Vitamin D supplement doses for the prevention of falls in older adults ([87]).
- The SPRINT trial on safety and efficacy of neublastin in painful lumbosacral radiculopathy ([88]).
- SARC009: A Phase II study in patients with previously treated, high-grade, advanced sarcoma ([89]).
- The SHINE clinical trial for hyperglycaemia in stroke patients ([90,91]).
- The EPAD project in neurology ([92]).
- The BATTLE and BATTLE-2 trials for lung cancer ([93,94]).
- The I-SPY 2 platform for breast cancer chemotherapy ([95]; see also [96,97,98]).
- A study on Lemborexant, for the treatment of insomnia disorder ([99]).
- A Phase I non-randomized trial of a combination therapy in patients with pancreatic adenocarcinoma ([100]).
- A first-in-human study of RG7342 for the treatment of schizophrenia in healthy male subjects ([101]).
- A newly started Phase II trial in Japan for sarcoma ([102]) also shows the utility of a Bayesian adaptive design.
- A Bayesian response-adaptive trial in tuberculosis is the endTB trial ([103]).
- Acute Stroke Therapy by Inhibition of Neutrophils (ASTIN) was a Bayesian adaptive phase 2 dose-response study to establish whether UK-279,276 improves recovery in acute ischemic stroke. The adaptive design facilitated early termination for futility ([104]).

## 7. Controversies

There is an on-going debate on adaptive designs in clinical trials. The criticisms are not addressed specifically to Bayesian methods, nor to the general class of adaptive designs, but circle around the value of response-adaptive randomization in clinical trials. The much criticized Michigan ECMO trial (see [105,106]), which took place 35 years ago, is often advocated to discourage the use of response-adaptive randomization in practice. It is not an aim of this paper to get involved in these controversies: among the rest, there are still too many open methodological questions surrounding the use of adaptive designs and their inference, whether Bayesian or not. On the other hand, it would be unfair not to mention the existence of this debate.

First of all the usefulness of adaptive randomized design is questioned: Korn and Freidlin [107] and Yuan and Yin [108] suggest that in the binary case outcome-adaptive randomization might not have substantial advantages over fixed-ratio when the response rate of the experimental treatment is not substantially higher than that of the standard treatment. Berry [109], however, disproves their conclusion. Korn and Freidlin [110] examine further the negative effects of response-adaptive randomization in some well-known trials. Lee, Chen and Yin [111] give a balanced view, as the result of extended simulations.

Another issue is whether adaptive randomization designs are ethical: Hey and Kimmelman [112] and Wathen and Thall [113] argue that the chance that adaptive random allocation will assign more patients to an inferior arm is too high. Other authors (Wason et al. [114]) worry that these designs may be too complex for use and that standard analysis methods of analyzing results from adaptive trials are valid only asymptotically. Another main concern is bias from temporal trends ([115]).

In conclusion, Thall, Fox and Wathen [116] state that adaptive randomization produces inferential problems that decrease potential benefit to future patients, and may decrease benefit to patients enrolled in the trial. These problems should be weighed against its putative ethical benefit. For randomized comparative trials to obtain confirmatory comparisons, designs with fixed randomization probabilities and group sequential decision rules appear to be preferable to adaptive randomization, scientifically and ethically.

Whereas this controversy has been useful to learn how response-adaptive randomization might be used appropriately, for instance by introducing a burn-in period of equal randomization in some adaptive trials, the class of adaptive designs is really vast, so any discussion should focus around the value of a specific type of adaptive design in the specific context for which it is being proposed. Unfortunately, the arguments are often generic and not applied to the special features of adaptive procedures, as also Villar et al. [117] underline. In particular, as mentioned in Section 2 about the choice of the utility function, priorities about the very purpose or purposes of the experiment should be made clear in advance.

## 8. Conclusions

“*Bayesian adaptive clinical trials: a dream for statisticians only*?” asks Chevret [44]. Clearly, Bayesian adaptive experiments are not easy to design, let alone to implement. For a start, elicitation of a prior is not a simple matter. In clinical trials it is generally assumed to be based on historical data. In their book ([1]) Spiegelhalter, Abrams and Myles recommend attempting both an “enthusiastic” and a “skeptical” prior. On the other hand, Bayesian statistics exercises greater appeal than frequentist on most applied researchers, and the same can be said of adaptive design rules. This explains why the presence of Bayesian and adaptive design methods combined together has become massive in the biostatistical literature, notwithstanding the fact that adaptive algorithms are more complex than non-adaptive.

It is this author’s opinion that although there is a widespread consensus that the Bayesian and the adaptive approaches to design go very well together, the field is still rather fragmented. The development has taken place in a relatively short time and Bayesian adaptive designs are still awaiting in-depth investigation. It is a sad state of affairs that in general there is no sounder way to evaluate the performance of Bayesian (and non-Bayesian) designs other than by computer simulations. Often the simulation scenarios are chosen on the basis of the researchers’ personal preferences, so the conclusions may be debatable.

The book by Yin [118] is a thorough presentation of both Bayesian and frequentist adaptive methods in clinical trial design, but the two approaches are based on fundamentally different paradigms and a comparison of Bayesian and non-Bayesian designs is possible only in restricted cases. As an example, when several experimental treatments are available for testing, Wason and Trippa [119] compare Bayesian adaptive randomization, which allocates a greater proportion of future patients to treatments that have performed well, to multi-arm multi-stage designs, which use pre-specified stopping boundaries to determine whether experimental treatments should be dropped. The authors show that in this case both are efficient, but neither is superior: it depends on the true state of nature.

In conclusion, it is worth quoting the words of Stallard et al. [120]: “*Bayesian adaptive methods are often more bespoke than frequentist approaches… They require more design work than the use of a more standard frequentist method but can be advantageous in that design choices and their consequences are considered carefully*”.
